# TNF-*α* stimulates System A amino acid transport in primary human trophoblast cells mediated by p38 MAPK signaling

**DOI:** 10.14814/phy2.12594

**Published:** 2015-10-27

**Authors:** Irving L M H Aye, Thomas Jansson, Theresa L Powell

**Affiliations:** 1 Division of Reproductive Sciences, Department of Obstetrics and Gynecology, University of Colorado Anschutz Medical CampusAurora, Colorado; 2 Section of Neonatology, Department of Pediatrics, University of Colorado Anschutz Medical CampusAurora, Colorado

**Keywords:** Cytokines, inflammation, maternal–fetal exchange, nutrient transport, placenta

## Abstract

Maternal obesity and gestational diabetes mellitus (GDM) increase the risk of delivering infants that are large for gestational age with greater adiposity, who are prone to the development of metabolic disease in childhood and beyond. These maternal conditions are also associated with increased levels of the proinflammatory cytokine TNF-*α* in maternal tissues and the placenta. Recent evidence suggests that changes in placental amino acid transport contribute to altered fetal growth. TNF-*α* was previously shown to stimulate System A amino acid transport in primary human trophoblasts (PHTs), however the molecular mechanisms remain unknown. In this study, we tested the hypothesis that TNF-*α* regulates amino acid uptake in cultured PHTs by a mitogen-activated protein kinase (MAPK)-dependent mechanism. Treatment of PHTs with TNF-*α* significantly increased System A amino acid transport, as well as Erk and p38 MAPK signaling. Pharmacological antagonism of p38, but not Erk MAPK activity, inhibited TNF-*α* stimulated System A activity. Silencing of p38 MAPK using siRNA transfections prevented TNF-*α* stimulated System A transport in PHTs. TNF-*α* significantly increased the protein expression of System A transporters SNAT1 and SNAT2, but did not affect their mRNA expression. The effects of TNF-*α* on SNAT1 and SNAT2 protein expression were reversed by p38 MAPK siRNA silencing. In conclusion, TNF-*α* regulates System A activity through increased SNAT1 and SNAT2 transporter protein expression in PHTs. These findings suggest that p38 MAPK may represent a critical mechanistic link between elevated proinflammatory cytokines and increased placental amino acid transport in obese and GDM pregnancies associated with fetal overgrowth.

## Introduction

Maternal obesity and gestational diabetes mellitus (GDM) create an intrauterine environment that promotes fetal overgrowth (Group HSCR, 2008; Catalano et al. 2009), altered body composition (Catalano et al. 2009; Uebel et al. 2014), and increased risk of childhood obesity (Crume et al. 2011). As the interface between maternal and fetal circulations, the placenta represents a vital determinant of fetal growth through its role in nutrient delivery to the fetus. Indeed, altered placental nutrient transport, in particular amino acid transport, is a common pathway which leads to pathological fetal growth resulting in a fetus that is either large for gestational age with increased placental amino acid transport (Jansson et al. 2013) or growth restricted in cases of decreased amino acid transport (Glazier et al. 1997; Jansson et al. 1998). It is now increasingly recognized that placental nutrient transport function is highly influenced by the maternal environment (Jansson et al. 2012; Aye et al. 2013b; Gaccioli et al. 2013; Diaz et al. 2014).

Maternal obesity and GDM are conditions associated with chronic low-grade inflammation, resulting in increased levels of proinflammatory cytokines IL-6 and TNF-*α* in the maternal circulation (Ategbo et al. 2006; Aye et al. 2014b) and the placenta (Roberts et al. 2009; Oliva et al. 2012). IL-6 and TNF-*α* have been previously shown to stimulate System A amino acid transporter activity in cultured primary human trophoblast cells (PHTs) of the term placenta (Jones et al. 2009) as well as in hepatocyte cell lines (Watkins et al. 1994; Goenner et al. 1997). Furthermore, placental System A activity is positively correlated with birth weight in women across a range of body mass indices (Jansson et al. 2013), suggesting a link between maternal adiposity, systemic inflammation, placental nutrient transport, and birth weight.

System A amino acid transporters mediate sodium-dependent uptake of small, neutral amino acids such as alanine, serine, and glutamine (Christensen et al. 1965). There are three System A isoforms, sodium-coupled neutral amino acid transporter (SNAT) 1, SNAT2, and SNAT4, encoded by the genes *Slc38a1*, *Slc38a2*, and *Slc38a4*, respectively (Broer 2014). All three SNAT isoforms are expressed in the maternal-facing microvillus membrane of the human placenta (Hatanaka et al. 2000; Wang et al. 2000; Desforges et al. 2009). SNAT1 and SNAT2 exhibit similar properties with regard to substrate specificities and affinities, whereas SNAT4 has a lower affinity for neutral amino acids and also transports cationic amino acids (Hatanaka et al. 2001; Kudo and Boyd 2002). Importantly, these transporters establish an intracellular gradient of neutral amino acids, which can then be used to drive the uptake of essential amino acids such as leucine through exchange mechanisms mediated by System L transporters (Verrey 2003).

In our previous study, IL-6 was shown to stimulate System A activity in primary human trophoblasts (PHTs) through STAT3-dependent regulation of the System A transporter SNAT2 (Jones et al. 2009). Additionally, System A transport activity in PHTs is also activated by TNF-*α* (Jones et al. 2009), although the underlying molecular mechanisms are currently unknown. In this study, we sought to identify the cellular signaling mechanisms by which TNF-*α* regulates System A amino acid transport. Mitogen-activated protein kinases (MAPKs) respond to a diverse array of stimuli including proinflammatory cytokines and growth factors, and regulate a number of cellular metabolic processes. There are three subfamilies of MAPKs that are activated by both inflammatory and mitogenic signals, extracellular signal-regulated kinases (Erk), c-Jun N-terminal kinases (JNK), and p38 MAPK. The objective of this study was to test the hypothesis that TNF-*α* regulates amino acid uptake in cultured PHT cells through a MAPK-dependent mechanism.

## Materials and Methods

### Study subjects and tissue collection

Human placental tissue samples were collected from a total of 25 healthy women with normal term pregnancies who were scheduled for delivery by elective Cesarean section following written informed consent. Placental tissues were coded and de-identified relevant medical information was provided through the repository. This study was approved by the Colorado Multiple Institutional Review Board (COMIRB-14-1073). The early pregnancy (<14 weeks gestation) body mass index of the women included in this study ranged from 20.3 to 29.8.

### Primary human trophoblast cell culture and treatments

Placental tissue was transported to the laboratory within 15 min of delivery, and PHT cells were isolated by trypsin digestion and Percoll purification as originally described (Kliman et al. 1986) with modifications (Roos et al. 2009; Aye et al. 2013a, 2014a). Briefly, approximately 40 g of villous tissue was dissected free of decidua and blood vessels, washed in phosphate-buffered saline (PBS), and digested in trypsin (0.25%, Invitrogen, Carlsbad, CA) and DNAse I (Sigma-Aldrich, St. Louis, MO). Digests were then poured through 70-*μ*m cell filters (BD Bioscience, San Jose, CA) and cytotrophoblast cells purified over a discontinuous 10–70% Percoll gradient centrifugation. Cells which migrated between 35% and 55% Percoll layers were collected and cultured in 1:1 mixture of Dulbecco’s modified Eagle’s medium (DMEM, Sigma-Aldrich) and Ham’s F-12 nutrient mixture (Invitrogen) containing 10% fetal bovine serum (FBS, Atlanta Biologicals, Lawrenceville, GA), 50 *μ*g/mL gentamicin, 60 *μ*g/mL benzyl penicillin, and 100 *μ*g/mL streptomycin (Sigma-Aldrich), and incubated in a 5% CO_2_ humidified atmosphere at 37°C. Following 18 h of culture, attached PHT cells were washed twice in warmed Dulbecco’s PBS and culture media was changed daily over 90 h. Functional and expression analyses were performed at 90 h when the cultured cytotrophoblast cells have differentiated into syncytiotrophoblasts.

At 66 h (total culture time), PHT cells were treated with recombinant human TNF-*α* (10 pg/mL, Sigma-Aldrich) or vehicle control (PBS) in culture media containing 1% FBS as described previously (Aye et al. 2013a). We have chosen this concentration of TNF-*α* because it is within the physiological range of maternal circulating levels at term in normal and GDM women (Kirwan 2002; Cseh et al. 2004; Altinova et al. 2007; Saucedo et al. 2011), and produces a consistent effect on amino acid uptake. For pharmacological antagonism of p38 or Erk MAPK activity, PHT cells were treated with the p38 MAPK inhibitor SB203580, 10 μmol/L (SB, Cell Signaling Technology) or Erk MAPK inhibitor U0126, 0.1 μmol/L (U0, Cell Signaling Technology) for 30 min prior to TNF-*α* stimulation. All experiments were terminated at 90 h of culture. At this time, cell lysates were processed for RNA extraction or protein lysates, and amino acid uptake or cell viability assays were performed.

### Small interfering RNA (siRNA) transfection

Cells were plated either at 2.75 million per 35-mm dish for RNA and protein analyses, or 2 million per well in a six-well plate for amino acid transport assays. Following 18 h of culture, PHT cells were transfected with 100 nmol/L of siRNA targeting p38 MAPK (Sigma-Aldrich, SASI_Hs01_00018467) or nontargeting Scrambled (Scr) siRNA (SIC001, Sigma-Aldrich) using Dharmafect2 transfection reagent (ThermoScientific, Waltham, MA, USA) according to the manufacturer’s protocol and as reported previously (Aye et al. 2014a).

### Biochemical characterization and viability of primary human trophoblast cells

To determine the effects of siRNA transfection or pharmacological treatments on trophoblast differentiation, human chorionic gonadotropin secretion into cell culture media was measured using a commercial ELISA kit according to the manufacturer’s instructions (IBL-America, Minneapolis, MN).

The viability of PHTs following siRNA transfection or pharmacological treatments was determined by the ability of cultured cells to metabolize 4,5-dimethylthiazol-2-yl-2,5-diphenyltetrazolium bromide (MTT, Sigma-Aldrich) (van Meerloo et al. 2011). Briefly, PHT cells plated in 96-well plates were incubated with 1 mg/mL of MTT reconstituted in PBS for 4 h at 37°C, lysed with 10% SDS, and absorbance read at 570 nm.

### Amino acid transport

System A amino acid transport was determined by measuring Na^+^-dependent uptake of ^14^C-methyl-aminoisobutyric acid (MeAIB) and System L transport activity measured as 2-amino-2-norbornane-carboxylic acid (BCH)-inhibitable uptake of ^3^H-leucine (Leu), as described previously (Roos et al. 2009). Following treatment of PHTs as indicated above, cells plated in triplicate were washed three times in Tyrode’s salt solution with or without Na^+^ (iso-osmotic choline replacement) prewarmed to 37°C. Cells were then incubated with Tyrode’s salt solution (with Na^+^ or Na^+^-free with addition of 1 mmol/L BCH) containing ^14^C-MeAIB (final concentration 20 μmol/L) and ^3^H-Leu (final concentration 12.5 nmol/L) for 8 min. Transport was terminated by washing cells three times with ice-cold Tyrode’s salt solution without Na^+^. Cells were then lysed in distilled water and the water was counted in a liquid scintillation counter. Protein content of lysed cells was determined using the Lowry method (Lowry et al. 1951). Transporter-mediated uptakes were calculated by subtracting uptake in Na^+^-free/BCH buffer (nonmediated uptake) from uptake in Na^+^-containing buffer (total uptake) and transport activity is expressed as pmol per mg of protein per minute (pmol/mg/min).

### Reverse transcription and quantitative polymerase chain reaction (Q-PCR)

Total RNA was extracted using TRIzol Reagent (Life Technologies, Carlsbad, CA). cDNA synthesis was performed using the High-Capacity RNA-to-cDNA kit (Life Technologies). Q-PCR for SNAT1, SNAT2, SNAT4, succinate dehydrogenase complex subunit A (SDHA), and TATA box-binding protein (TBP) was performed in triplicate on 0.2 *μ*g of total RNA reverse transcribed into cDNA using SYBR Select Master Mix (Life Technologies). PCR amplification and detection were performed on a Quant Studio 6 Flex Real-Time PCR system (Life Technologies) using the following primers: SNAT1 – forward 5′–GTGTATGCTTTACCCACCATTGC–3′ and reverse 5′–GCACGTTGTCATAGAATGTCAAGT–3′; SNAT2 – forward 5′–ACGAAACAATAAACACCA-CCTTAA–3′ and reverse 5′–AGATCAGAATT GGCACAGCATA–3′; SNAT4 – forward 5′–TTGCCGCCCTCTTTGGTTAC–3′ and reverse 5′–GAGGACAATGGGCACAGTTAGT–3′; SDHA – forward 5′–TACAAGGTGCGGATTGATGA–3′ and reverse 5′–CACAGTCAGCCTCGTTCAAA–3′; and TBP – forward 5′–GTTCTGGGAAAATGGTGTGC–3′ and reverse 5′–GCTGGAAAACCCAACTTCTG–3′. Amplification of a single product was confirmed by melting curve analysis. The amplified transcripts were quantified using the relative standard curve method and normalized to the geometric mean of SDHA and TBP.

### Western blot analyses

Cells were harvested in radioimmunoprecipitation (RIPA) buffer (50 mmol/L Tris HCl, pH 7.4, 150 mmol/L NaCl, 0.1% SDS, 0.5% Na-deoxycholate, and 1% Triton X-100) containing protease inhibitors and phosphatase inhibitor cocktail 1 and 2 (1:100, Sigma-Aldrich). Protein concentrations were determined using the bicinchoninic acid assay as per the manufacturer’s instructions using bovine serum albumin (BSA) as the standard (Thermo Scientific, Rockford, IL).

Equal amounts of protein (3 *μ*g) were loaded into each well and separated on Any KD Mini-Protean Tris Glycine Pre-cast gels (BioRad, Hercules CA). Separated proteins were then transferred onto polyvinylidene difluoride membranes (Thermo Scientific) and blocked with 5% blotting-grade blocker (BioRad) for 1 h. After washing in Tris-buffered saline containing 0.1% Tween (TBS-T), membranes were incubated in primary antibodies overnight at 4°C in TBS-T containing 2.5% BSA. Primary antibodies were diluted as follows: rabbit anti-phospho-Erk1/2 (Thr202/Tyr204), phospho-JNK1/2/3 (Thr183/Tyr185), phospho-p38 MAPK (Thr180/Tyr182), Erk, JNK, p38 MAPK, SNAT1, SNAT2, and SNAT4 at 1 *μ*g/mL; and mouse anti-*β*-actin at 0.2 *μ*g/mL.

The membranes were then washed and incubated with peroxidase-conjugated goat anti-rabbit (1:2000) or anti-mouse antibody (1:5000) in TBS-T with 2% BSA for 2 h at room temperature and visualized by enhanced chemiluminescence using ECL Western Blotting Substrate (Pierce). Resultant images were captured on a G:Box ChemiXL1.4 (Syngene, Cambridge, UK) and bands quantified using Image J software (imagej.nih.gov). Target protein expression was normalized to *β*-actin expression. For each target protein, the mean density of the control sample bands was assigned an arbitrary value of 1. To determine whether the treatments influenced *β*-actin expression, membranes were also stained for total protein using Amido Black Stain (Sigma) and target protein expression quantified as described previously (Lanoix et al. 2012). Results corrected for *β*-actin and total protein staining using Amido Black stain were not different (data not shown) and therefore only data obtained with *β*-actin normalization are given.

All antibodies were obtained from Cell Signaling Technology, with the exception of the SNAT isoforms. SNAT1 antibody was obtained from Sigma-Aldrich. SNAT2 antibody was generated as described in Ling et al. (Ling et al. 2001) and was received as a generous gift from Dr. V. Ganapathy and Dr. P. Prasad at the University of Georgia, Augusta. SNAT4 antibodies were produced in rabbits using the epitope YGEVEDELLHAYSKV in human SNAT4 (Eurogentec, Seraing, Belgium) and previously characterized in placental tissues and trophoblast cells (Desforges et al. 2009; Roos et al. 2009).

### Data presentation and statistical analysis

All studies were repeated in primary cultures from 4 to 9 different placentas. Data are presented as mean + SEM. Statistical significance was determined either by Student’s *t*-test or by repeated measures ANOVA followed by Bonferroni post hoc test. *P* < 0.05 was considered significant. Statistical analysis and graph plotting were performed using Prism 5 software (Graph Pad, La Jolla, CA).

## Results

### TNF-*α* stimulates System A amino acid transport

**Figure 1:**
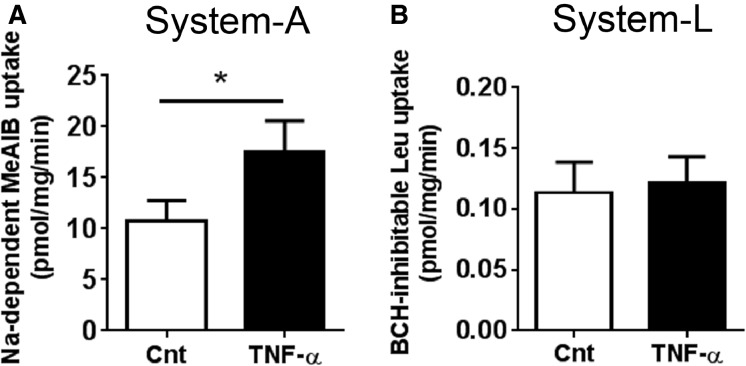
System A and System L amino acid transport activity in TNF-*α*-treated primary human trophoblasts. (A) System A and (B) System L transport activity as measured by MeAIB and leucine uptake, respectively, after exposure to TNF-*α* (10 pg/mL) for 24 h. Data are represent as mean + SEM, *n* = 6. *P < 0.05. Cnt, control; TNF-*α*, tumor necrosis factor-alpha.

System A activity was increased by approximately 50% following stimulation with 10 pg/mL of TNF-*α* (Fig.1A). This concentration is within the reported range for circulating concentrations of TNF-*α* (1–20 pg/mL) in normal healthy pregnant women and women with GDM (Cseh et al. 2002; Kirwan 2002; Altinova et al. 2007; Saucedo et al. 2011). System L-mediated uptake of leucine, on the other hand, was not affected by TNF-*α* (Fig.1B). These findings are consistent with previous reports demonstrating TNF-*α* regulation of System A, but not System L uptake activity in PHTs (Jones et al. 2009). On the basis of these findings, we have focused our studies on System A activity.

### Activation of MAPK pathways by TNF-*α* treatment

**Figure 2:**
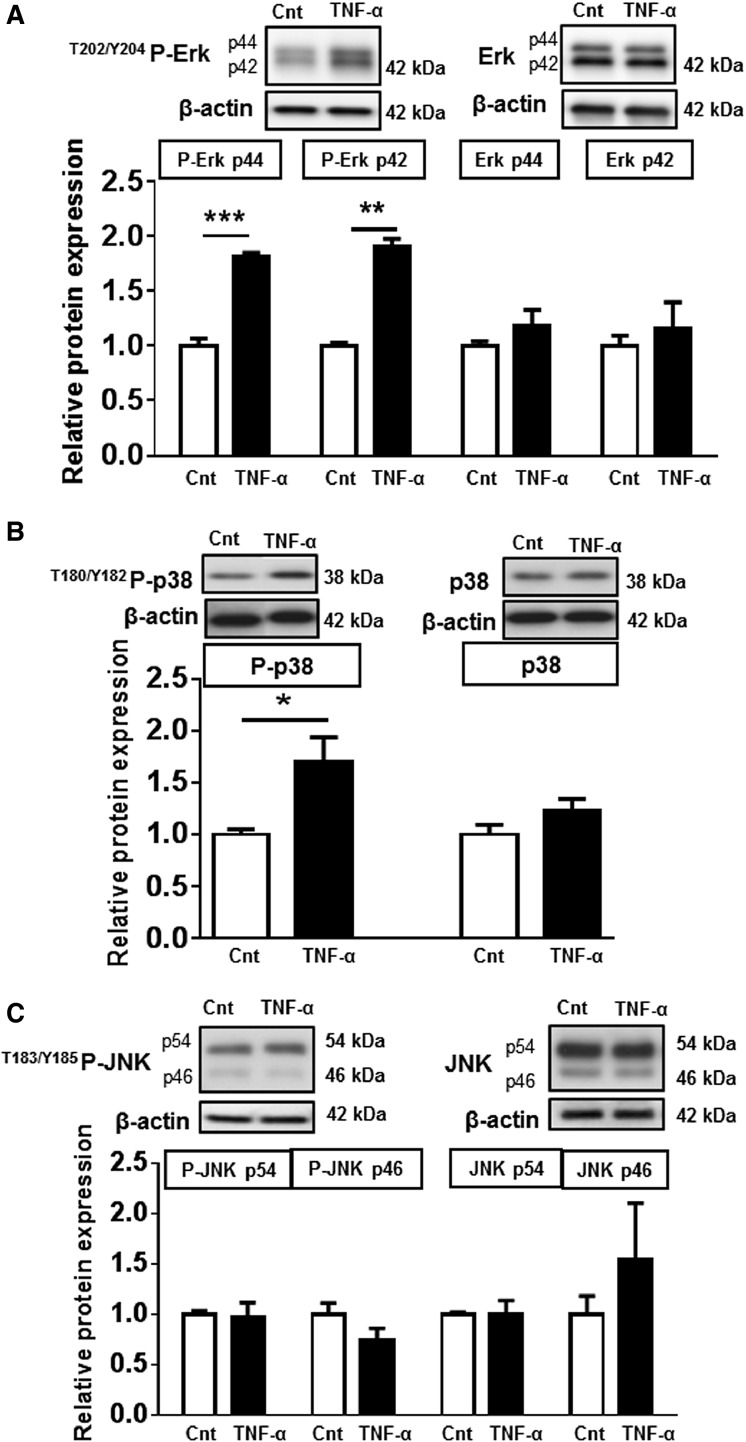
Activation of mitogen-activated protein kinase (MAPK) pathways following TNF-*α* exposure. Phosphorylated and total protein expression of (A) Erk, (B) p38, and (C) JNK MAPK in primary human trophoblasts after exposure to TNF-*α* (10 pg/mL). Data are presented as mean + SEM, *n* = 4. *P < 0.05, **P < 0.01, ***P < 0.001. Cnt, control; TNF-*α*, tumor necrosis factor-alpha.

In order to identify the mechanism(s) by which TNF-*α* regulates System A transport, we determined the effect of TNF-*α* on MAPK signaling activity. Treatment with TNF-*α* significantly increased the phosphorylation of Erk (Fig.2A) and p38 (Fig.2B), but not JNK MAPK (Fig.2C). TNF-*α* did not influence the total expression of Erk, p38, or JNK-MAPK proteins. On the basis of these findings, we targeted Erk and p38 MAPK to determine the involvement of these pathways in regulating trophoblast System A amino acid transport in response to TNF-*α*.

### Pharmacological antagonism of p38 MAPK prevents TNF-*α*-mediated System A transport

**Figure 3:**
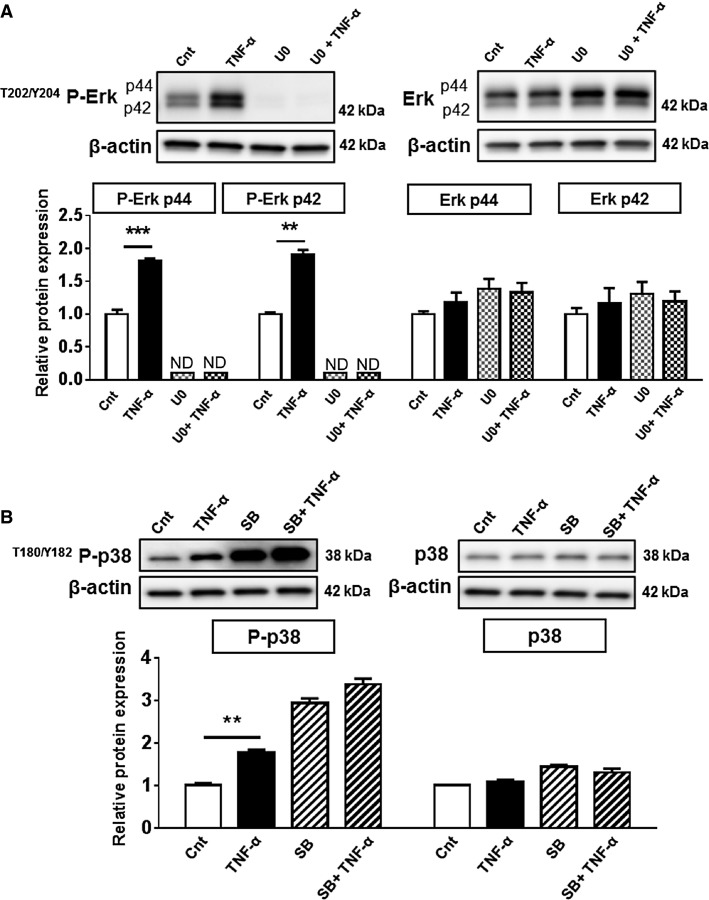
Pharmacological antagonism of Erk and p38 MAPK activity. Primary human trophoblasts were treated with the pharmacological antagonists U0126 (Erk inhibitor, 0.1 μmol/L) or SB203580 (p38 inhibitor, 10 μmol/L) 30 min prior to treatment with TNF-*α* (10 pg/mL) for 24 h. Phosphorylated and total protein expression of (A) Erk and (B) p38 MAPK. Data are presented as mean + SEM, *n* = 4. *P < 0.05, **P < 0.01, ***P < 0.001. ND, not detectable; Cnt, control; TNF-*α*, tumor necrosis factor-alpha; U0, U0126; SB, SB203580.

**Figure 4:**
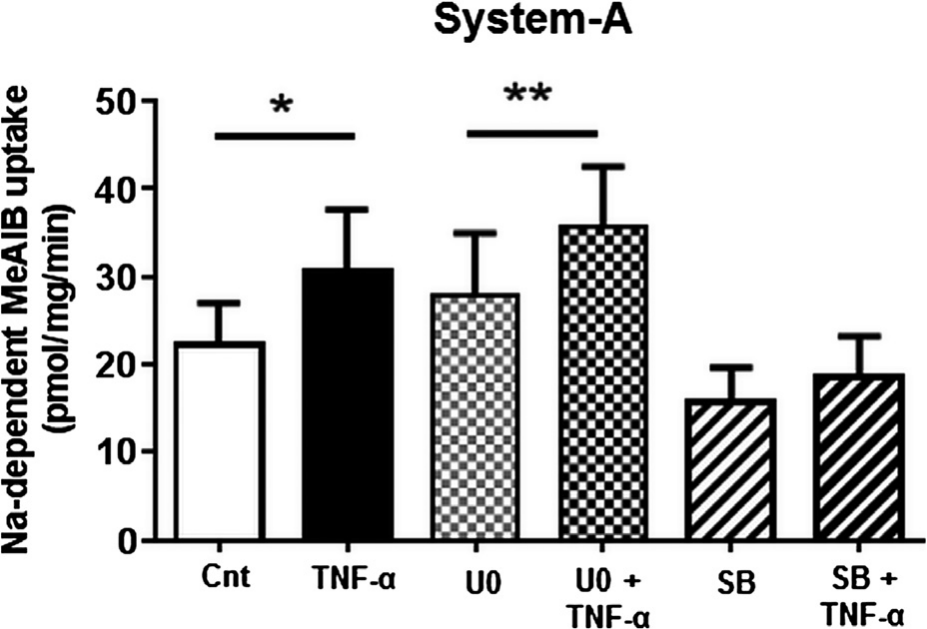
System A uptake activity following treatment with TNF-*α* and Erk/p38 MAPK antagonists. Na^+^-dependent MeAIB uptake was measured in primary human trophoblasts following treatment with pharmacological antagonists U0126 (Erk inhibitor, 0.1 μmol/L) or SB203580 (p38 inhibitor, 10 μmol/L) MAPK, 30 min prior to exposure to TNF-*α* (10 pg/mL) for 24 h. Data are presented as mean + SEM, *n* = 9. *P < 0.05, **P < 0.01. Cnt, control; TNF-*α*, tumor necrosis factor-alpha.

Inhibition of Erk activity was achieved by targeting the upstream Erk kinases MEK1 and MEK2 using U0126 (Favata et al. 1998). U0126 significantly inhibited both basal and TNF-*α*-dependent Erk phosphorylation (Fig.3A). However, inhibition of Erk activity did not prevent TNF-*α*-mediated increase in System A activity (Fig.4). SB203580 inhibits p38 MAPK catalytic activity by binding to the ATP-binding site, but does not inhibit the phosphorylation of p38 MAPK by upstream kinases (Kumar et al. 1999). Consistent with these reports, SB203580 treatment did not decrease p38 MAPK phosphorylation, but instead increased its phosphorylation (Fig.3B). Nevertheless, SB203580 prevented further p38 MAPK phosphorylation when treated with TNF-*α* (Fig.3B). Inhibition of p38 MAPK activity by SB203580 also inhibited TNF-*α*-mediated System A transport (Fig.4), suggesting that p38 MAPK activation is required for TNF-*α*-dependent increase in System A activity. Treatment with the pharmacological antagonists did not impair MTT cell viability or trophoblast differentiation as determined by human chorionic gonadotropin (hCG) secretion (data not shown).

### p38 MAPK silencing inhibits TNF-*α* stimulation of System A transporter protein expression and activity

**Figure 5:**
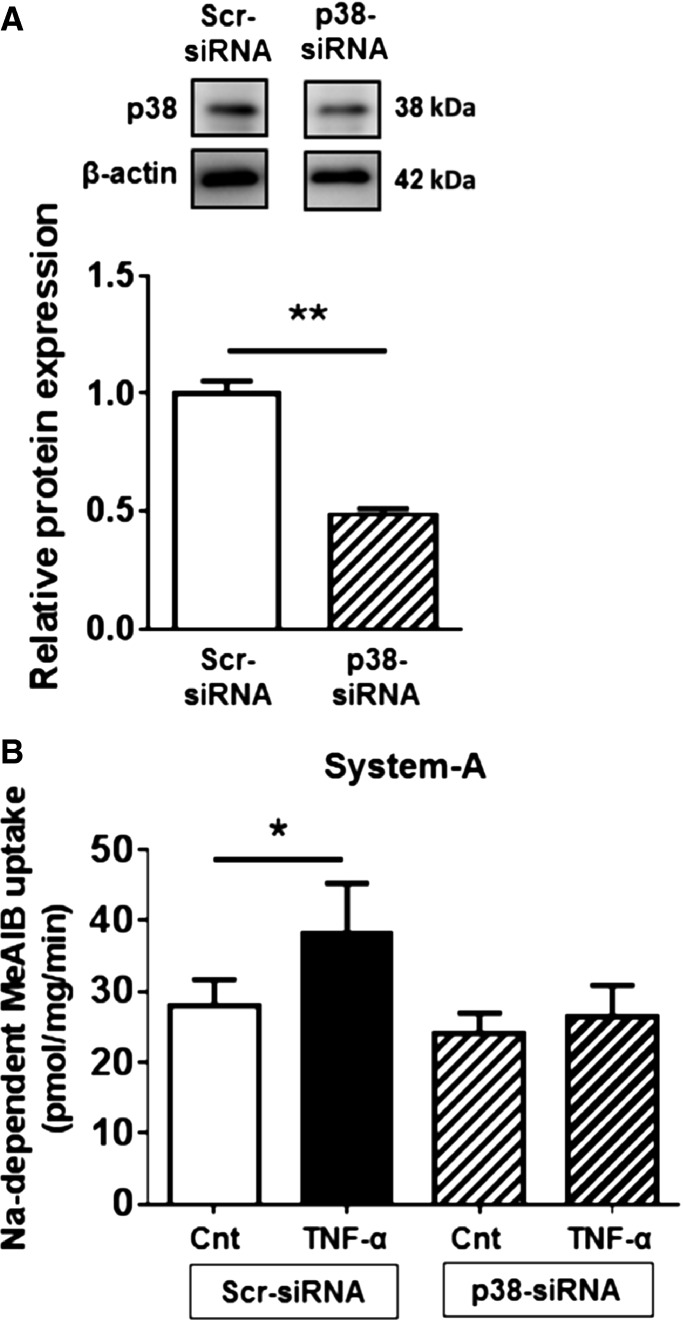
Effects of p38 MAPK silencing on System A transport activity. Primary human trophoblasts were transfected with p38 MAPK siRNA or scrambled siRNA controls. (A) p38 MAPK protein expression in Scr siRNA or p38 siRNA-transfected cells at 90 h of culture. Data are presented as mean + SEM, *n* = 4. (B) Na^+^-dependent MeAIB uptake (System A) activity was measured in Scr siRNA or p38 siRNA-transfected cells with/without TNF-*α* (10 pg/mL) treatment. Data are presented as mean + SEM, *n* = 5. *P < 0.05, **P < 0.01. Cnt, control; TNF-*α*, tumor necrosis factor-alpha; Scr, scramble; p38, p38 MAPK.

To further establish the role of p38 MAPK in regulating System A amino acid transport, we silenced p38 MAPK expression using RNA interference. Compared to scrambled siRNA, transfection with p38 siRNA reduced p38 MAPK protein expression by approximately 50% (Fig.5A). In PHT cells transfected with scrambled siRNA, TNF-*α* stimulated System A transport activity, whereas knockdown of p38 MAPK inhibited TNF-*α*-mediated transport (Fig.5B). siRNA transfections did not influence cell viability or hCG secretion (data not shown).

**Figure 6:**
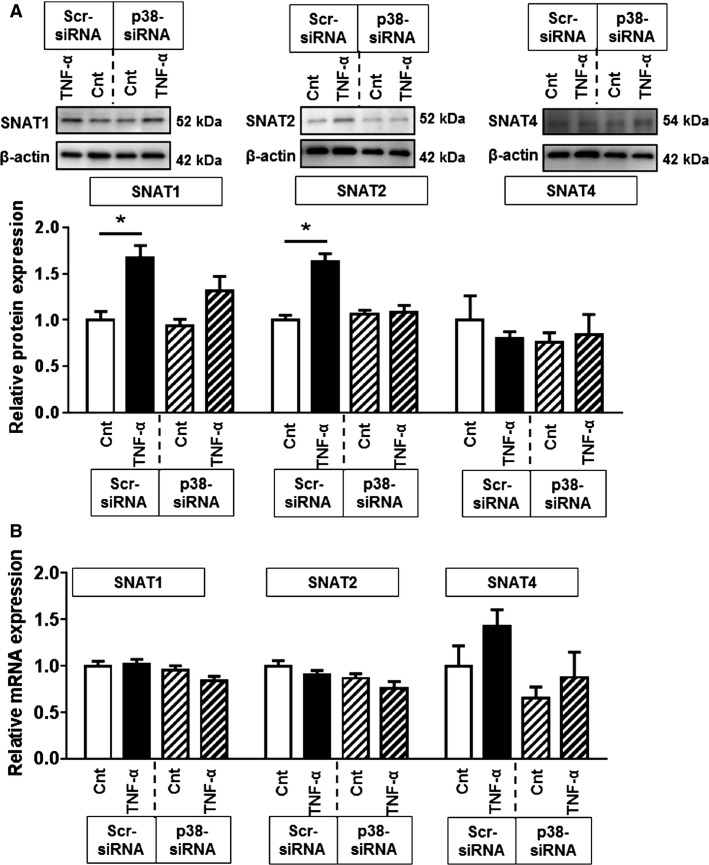
Regulation of SNAT1, SNAT2, and SNAT4 protein and mRNA expression by p38 MAPK. Primary human trophoblasts were transfected with p38 MAPK siRNA or scrambled siRNA controls with/without TNF-*α* (10 pg/mL) exposure for 24 h. (A) SNAT1, SNAT2, and SNAT4 protein expression normalized to *β*-actin. Data are presented as mean + SEM, *n *= 4. (B) mRNA expression of SNAT1, SNAT2, and SNAT4 normalized to the geometric mean of SDHA and TBP mRNA. Data are presented as mean + SEM, *n* = 6. *P < 0.05. Cnt, control; TNF-*α*, tumor necrosis factor-alpha; Scr, scramble; p38, p38 MAPK.

We then determined if the effects of p38 MAPK silencing were associated with decreased expression of System A amino acid transporter isoforms SNAT1, SNAT2, and SNAT4. TNF-*α* significantly increased SNAT1 and SNAT2 protein expression, but did not affect SNAT4 protein (Fig.6A). Silencing of p38 MAPK attenuated TNF-*α*-mediated increase in SNAT1 protein, whereas the increase in SNAT2 protein expression was completely prevented following p38 MAPK knockdown (Fig.6A). p38 MAPK silencing did not affect the basal protein expression of the SNAT isoforms (Fig.6A).

Interestingly, TNF-*α* stimulation did not alter the mRNA expression of SNAT1, SNAT2, or SNAT4 (Fig.6B). Moreover, p38 MAPK silencing also did not affect the mRNA expression of System A amino acid transporter isoforms (Fig.6B). Taken together, these findings strongly suggest that TNF-*α* regulates System A transport activity through p38 MAPK-dependent regulation of SNAT1 and SNAT2 protein expression.

## Discussion

Metabolic disorders of pregnancy such as obesity and GDM are characterized by a chronic low-grade inflammatory environment (Pantham et al. 2015). Proinflammatory cytokines play an important role in regulating placental transport of nutrients including fatty acids (Lager et al. 2010) and amino acids (Jones et al. 2009). Hence, understanding the molecular mechanisms by which cytokines modulate nutrient transport may aid in the development of therapeutic strategies to reduce the incidence of fetal overgrowth in obese and GDM pregnancies, and thus reduce the burden of pediatric obesity and the development of adult metabolic diseases.

In this study, we provide mechanistic evidence that TNF-*α* regulation of System A amino acid transport is dependent on p38 MAPK. Consistent with previous reports, TNF-*α* stimulated System A activity in cultured PHTs but did not affect System L activity (Jones et al. 2009). Our previous study established the requirement of STAT3 in IL-6-dependent regulation of System A activity. STAT3 can be activated by cytokines such as IL-6, and hormones including leptin and insulin, and therefore lies at the nexus of cytokine and growth factor responses. Similarly, we propose that MAPKs may also represent such a system whereby growth factors and proinflammatory cytokines coordinate cellular metabolic processes.

Studies in nontrophoblast cells are consistent with a role for Erk and p38 MAPK in the regulation of amino acid transport. For example, the adaptive increase in System A transport activity following amino acid starvation requires Erk MAPK activation in human fibroblasts (Franchi-Gazzola et al. 1999; Lopez-Fontanals et al. 2003), and both Erk and p38 MAPK antagonists inhibited glutamate and glucose transport in intestinal cell lines (Gould et al. 1995; Meng et al. 2004). Moreover, our previous study showed that silencing of p38 MAPK inhibited insulin-stimulated System A activity in PHTs (Aye et al. 2014a). We therefore hypothesized that the increase in Erk and/or p38 MAPK would regulate the effects of TNF-*α* on System A transport activity in PHTs. While inhibition of Erk activity did not influence System A activity, p38 MAPK antagonism inhibited System A transport. In addition, silencing of p38 MAPK prevented the stimulatory effect of TNF-*α* on System A activity.

The effects of TNF-*α* and p38 MAPK on System A transport were associated with changes in the expression of SNAT1 and SNAT2 proteins but not their mRNA expression. In agreement with our findings, previous reports have also demonstrated SNAT2 regulation at the protein level. We recently showed that SNAT2 activity in PHTs is regulated at the posttranslational level by the mechanistic target of rapamycin (mTOR) (Rosario et al. 2013), and p38 MAPK has been shown to regulate the downstream activation of the mTOR target S6K1 (Casas-Terradellas et al. 2008). However, mTOR-dependent regulation of SNAT2 is mediated by trafficking of the transporter protein to the plasma membrane rather than total SNAT2 protein expression in cells (Rosario et al. 2013). Previous reports suggest that cellular levels of SNAT2 protein may be degraded by the ubiquitin proteasome system (Nardi et al. 2015), and p38 MAPK has been shown to promote protein stability by suppressing protein ubiquitination (Xie et al. 2012). Therefore, the possibility exists that p38 MAPK regulates SNAT2 protein stability in PHTs by inhibiting SNAT2 ubiquitination and proteasomal degradation.

TNF-*α* failed to stimulate System A activity in the presence of the p38 MAPK inhibitor SB203580, suggesting that TNF-*α* regulates this amino acid transport system mediated by p38 MAPK. Counterintuitively, SB203580 increased p38 MAPK phosphorylation, which has been reported in the literature previously (Kumar et al. 1999). For example, Kumar and coworkers showed a dose-dependent increase in p38 phosphorylation with SB203580 in a number of cell lines including human monocytic THP-1 cells, osteosarcoma MG63 and chondrocyte C20A4 cells (Kumar et al. 1999). Thus, the functional significance of phosphorylation of p38 MAPK at T180/Y182 is unclear. Importantly, however, using RNAi to knockdown p38 MAPK expression, we provide conclusive evidence that p38 MAPK mediates the effects of TNF-*α* on trophoblast amino acid transport.

The concentration of TNF-*α* used in this study reflects the upper range of circulating TNF-*α* levels in pregnant women at term (Cseh et al. 2002; Kirwan 2002; Altinova et al. 2007; Saucedo et al. 2011; Xu et al. 2014). Because the placenta abundantly produces cytokines, it is possible that the local concentrations of TNF-*α* in the intervillous space may be higher than systemic concentrations. However, we have chosen not to determine the effects of high TNF-*α* concentrations because it may trigger apoptosis (Smith et al. 2002) and/or impair trophoblast cell viability or endocrine function (Li et al. 1992; Pijnenborg et al. 2000), effects which are also likely to influence trophoblast System A activity.

The overall effects of inflammation on placental nutrient transport are complex and may vary on the degree and type of inflammation, the specific cytokines that are elevated, or even the type of transporter in question. Although our findings suggest that System A activity is stimulated by TNF-*α* or IL-6 (Jones et al. 2009) at concentrations characteristic of chronic metabolic disease (Xu et al. 2014; Pantham et al. 2015), in other situations such as infections or septic shock, the impact of acute inflammatory stress on placental nutrient transport may differ. For example, it has been proposed that the inflammatory response to placental malaria decreases System A transport activity and contributes to fetal growth restriction (Boeuf et al. 2013). High concentrations of TNF-*α* have also been reported to inhibit folic acid and methionine uptake in cultured trophoblasts (Araujo et al. 2013a,b). Furthermore, we recently reported that while IL-1*β* does not influence basal System A transport, it inhibited insulin-mediated System A activity (Aye et al. 2013a). On the other hand, IL-1*β* stimulated System L activity in PHTs (Aye et al. 2013a). From these studies, it is evident that complex interactions exist between cytokines and growth factors in regulating different placental nutrient transport systems.

Chronic low-grade inflammation associated with obesity and GDM may play an important role in the in utero programming of metabolic disease. This may, at least in part, be due to cytokine regulation of placental nutrient transport. However, the signaling pathways responsible have so far remained elusive. In this study, we have established p38 MAPK as a critical mediator of TNF-*α*-dependent System A amino acid transport in cultured PHTs. These in vitro findings provide mechanistic insight linking our previous observations in obese mothers of elevated maternal circulating levels of TNF-*α* (Aye et al. 2014b), increased placental p38 MAPK activity (Aye et al. 2014b), and increased amino acid transport associated with fetal overgrowth (Jansson et al. 2013).

## References

[b1] Altinova AE, Toruner F, Bozkurt N, Bukan N, Karakoc A, Yetkin I (2007). Circulating concentrations of adiponectin and tumor necrosis factor-alpha in gestational diabetes mellitus. Gynecol. Endocrinol.

[b2] Araujo JR, Correia-Branco A, Moreira L, Ramalho C, Martel F, Keating E (2013a). Folic acid uptake by the human syncytiotrophoblast is affected by gestational diabetes, hyperleptinemia, and TNF-alpha. Pediatr. Res.

[b3] Araujo JR, Correia-Branco A, Ramalho C, Goncalves P, Pinho MJ, Keating E (2013b). l-methionine placental uptake: characterization and modulation in gestational diabetes mellitus. Reprod Sci.

[b4] Ategbo JM, Grissa O, Yessoufou A, Hichami A, Dramane KL, Moutairou K (2006). Modulation of adipokines and cytokines in gestational diabetes and macrosomia. J. Clin. Endocrinol. Metab.

[b5] Aye IL, Jansson T, Powell TL (2013a). Interleukin-1beta inhibits insulin signaling and prevents insulin-stimulated system A amino acid transport in primary human trophoblasts. Mol. Cell. Endocrinol.

[b6] Aye IL, Powell TL, Jansson T (2013b). Review: adiponectin–the missing link between maternal adiposity, placental transport and fetal growth?. Placenta.

[b7] Aye IL, Gao X, Weintraub ST, Jansson T, Powell TL (2014a). Adiponectin Inhibits Insulin Function in Primary Trophoblasts by PPARalpha-Mediated Ceramide Synthesis. Mol. Endocrinol.

[b8] Aye IL, Lager S, Ramirez VI, Gaccioli F, Dudley DJ, Jansson T (2014b). Increasing maternal body mass index is associated with systemic inflammation in the mother and the activation of distinct placental inflammatory pathways. Biol. Reprod.

[b9] Boeuf P, Aitken EH, Chandrasiri U, Chua CL, McInerney B, McQuade L (2013). Plasmodium falciparum malaria elicits inflammatory responses that dysregulate placental amino acid transport. PLoS Pathog.

[b10] Broer S (2014). The SLC38 family of sodium-amino acid co-transporters. Pflugers Arch.

[b11] Casas-Terradellas E, Tato I, Bartrons R, Ventura F, Rosa JL (2008). ERK and p38 pathways regulate amino acid signalling. Biochim. Biophys. Acta.

[b12] Catalano PM, Presley L, Minium J, de Hauguel- Mouzon S (2009). Fetuses of obese mothers develop insulin resistance in utero. Diabetes Care.

[b13] Christensen HN, Oxender DL, Liang M, Vatz KA (1965). The use of *N*-methylation to direct route of mediated transport of amino acids. J. Biol. Chem.

[b14] Crume TL, Ogden L, Daniels S, Hamman RF, Norris JM, Dabelea D (2011). The impact of in utero exposure to diabetes on childhood body mass index growth trajectories: the EPOCH study. J. Pediatr.

[b15] Cseh K, Baranyi E, Melczer Z, Csakany GM, Speer G, Kovacs M (2002). The pathophysiological influence of leptin and the tumor necrosis factor system on maternal insulin resistance: negative correlation with anthropometric parameters of neonates in gestational diabetes. Gynecol. Endocrinol.

[b16] Cseh K, Baranyi E, Melczer Z, Kaszas E, Palik E, Winkler G (2004). Plasma adiponectin and pregnancy-induced insulin resistance. Diabetes Care.

[b17] Desforges M, Mynett KJ, Jones RL, Greenwood SL, Westwood M, Sibley CP (2009). The SNAT4 isoform of the system A amino acid transporter is functional in human placental microvillous plasma membrane. J. Physiol.

[b18] Diaz P, Powell TL, Jansson T (2014). The role of placental nutrient sensing in maternal–fetal resource allocation. Biol. Reprod.

[b19] Favata MF, Horiuchi KY, Manos EJ, Daulerio AJ, Stradley DA, Feeser WS (1998). Identification of a novel inhibitor of mitogen-activated protein kinase kinase. J. Biol. Chem.

[b20] Franchi-Gazzola R, Visigalli R, Bussolati O (1999). **Dall’Asta V, and Gazzola GC**. Adaptive increase of amino acid transport system A requires ERK1/2 activation. J. Biol. Chem.

[b21] Gaccioli F, Lager S, Powell TL, Jansson T (2013). Placental transport in response to altered maternal nutrition. J. Dev. Orig. Health Dis.

[b22] Glazier JD, Cetin I, Perugino G, Ronzoni S, Grey AM, Mahendran D (1997). Association between the activity of the system A amino acid transporter in the microvillous plasma membrane of the human placenta and severity of fetal compromise in intrauterine growth restriction. Pediatr. Res.

[b23] Goenner S, Cosson C, Boutron A, Legrand A, Moatti N (1997). Interleukin-1 beta and interleukin-6 stimulate 2-methylaminoisobutyric acid uptake in HepG2 cells. Int. J. Biochem. Cell Biol.

[b24] Gould GW, Cuenda A, Thomson FJ, Cohen P (1995). The activation of distinct mitogen-activated protein kinase cascades is required for the stimulation of 2-deoxyglucose uptake by interleukin-1 and insulin-like growth factor-1 in KB cells. Biochem. J.

[b25] Metzger BE, Lowe LP, Dyer AR, Trimble ER, Chaovarindr U, Coustan DR, Hadden DR, McCance DR, Hod M, McIntyre HD, Oats JJ, Persson B, Rogers MS, Sacks DA, Group HSCR (2008). Hyperglycemia and adverse pregnancy outcomes. The New England journal of medicine.

[b26] Hatanaka T, Huang W, Wang H, Sugawara M, Prasad PD, Leibach FH (2000). Primary structure, functional characteristics and tissue expression pattern of human ATA2, a subtype of amino acid transport system A. Biochim. Biophys. Acta.

[b27] Hatanaka T, Huang W, Ling R, Prasad PD, Sugawara M, Leibach FH (2001). Evidence for the transport of neutral as well as cationic amino acids by ATA3, a novel and liver-specific subtype of amino acid transport system A. Biochim. Biophys. Acta.

[b28] Jansson T, Scholtbach V, Powell TL (1998). Placental transport of leucine and lysine is reduced in intrauterine growth restriction. Pediatr. Res.

[b29] Jansson T, Aye IL, Goberdhan DC (2012). The emerging role of mTORC1 signaling in placental nutrient-sensing. Placenta.

[b30] Jansson N, Rosario FJ, Gaccioli F, Lager S, Jones HN, Roos S (2013). Activation of placental mTOR signaling and amino acid transporters in obese women giving birth to large babies. J. Clin. Endocrinol. Metab.

[b31] Jones HN, Jansson T, Powell TL (2009). IL-6 stimulates system A amino acid transporter activity in trophoblast cells through STAT3 and increased expression of SNAT2. Am. J. Physiol. Cell Physiol.

[b32] Kirwan JP, Hauguel-De Mouzon S, Lepercq J, Challier JC, Huston-Presley L, Friedman JE, Kalhan SC, Catalano PM (2002). TNF-alpha is a predictor of insulin resistance in human pregnancy. Diabetes.

[b33] Kliman HJ, Nestler JE, Sermasi E, Sanger JM, Strauss JF (1986). Purification, characterization, and in vitro differentiation of cytotrophoblasts from human term placentae. Endocrinology.

[b34] Kudo Y, Boyd CA (2002). Human placental amino acid transporter genes: expression and function. Reproduction.

[b35] Kumar S, Jiang MS, Adams JL, Lee JC (1999). Pyridinylimidazole compound SB 203580 inhibits the activity but not the activation of p38 mitogen-activated protein kinase. Biochem. Biophys. Res. Commun.

[b36] Lager S, Jansson N, Olsson AL, Wennergren M, Jansson T, Powell TL (2010). Effect of IL-6 and TNF-alpha on fatty acid uptake in cultured human primary trophoblast cells. Placenta.

[b37] Lanoix D, St-Pierre J, Lacasse AA, Viau M, Lafond J, Vaillancourt C (2012). Stability of reference proteins in human placenta: general protein stains are the benchmark. Placenta.

[b38] Li Y, Matsuzaki N, Masuhiro K, Kameda T, Taniguchi T, Saji F (1992). Trophoblast-derived tumor necrosis factor-alpha induces release of human chorionic gonadotropin using interleukin-6 (IL-6) and IL-6-receptor-dependent system in the normal human trophoblasts. J. Clin. Endocrinol. Metab.

[b39] Ling R, Bridges CC, Sugawara M, Fujita T, Leibach FH, Prasad PD (2001). Involvement of transporter recruitment as well as gene expression in the substrate-induced adaptive regulation of amino acid transport system A. Biochim. Biophys. Acta.

[b40] Lopez-Fontanals M, Rodriguez-Mulero S, Casado FJ, Derijard B, Pastor-Anglada M (2003). The osmoregulatory and the amino acid-regulated responses of system A are mediated by different signal transduction pathways. J. Gen. Physiol.

[b41] Lowry OH, Rosebrough NJ, Farr AL, Randall RJ (1951). Protein measurement with the Folin phenol reagent. J. Biol. Chem.

[b42] van Meerloo J, Kaspers GJ, Cloos J (2011). Cell sensitivity assays: the MTT assay. Methods Mol. Biol.

[b43] Meng Q, Epler MJ, Lin C, Karinch AM, Vary TC, Pan M (2004). Insulin-like growth factor-2 activation of intestinal glutamine transport is mediated by mitogen-activated protein kinases. J Gastrointest Surg.

[b44] Nardi F, Hoffmann TM, Stretton C, Cwiklinski E, Taylor PM, Hundal HS (2015). Proteasomal modulation of cellular SNAT2 (SLC38A2) abundance and function by unsaturated fatty acid availability. J. Biol. Chem.

[b45] Oliva K, Barker G, Riley C, Bailey MJ, Permezel M, Rice GE (2012). The effect of pre-existing maternal obesity on the placental proteome: two-dimensional difference gel electrophoresis coupled with mass spectrometry. J. Mol. Endocrinol.

[b46] Pantham P, Aye IL, Powell TL (2015). Inflammation in maternal obesity and gestational diabetes mellitus. Placenta.

[b47] Pijnenborg R, Luyten C, Vercruysse L, Van Assche JC, Keith FA (2000). Cytotoxic effects of tumour necrosis factor (TNF)-alpha and interferon-gamma on cultured human trophoblast are modulated by fibronectin. Mol. Hum. Reprod.

[b48] Roberts VH, Smith J, McLea SA, Heizer AB, Richardson JL, Myatt L (2009). Effect of increasing maternal body mass index on oxidative and nitrative stress in the human placenta. Placenta.

[b49] Roos S, Lagerlof O, Wennergren M, Powell TL, Jansson T (2009). Regulation of amino acid transporters by glucose and growth factors in cultured primary human trophoblast cells is mediated by mTOR signaling. Am. J. Physiol. Cell Physiol.

[b50] Rosario FJ, Kanai Y, Powell TL, Jansson T (2013). Mammalian target of rapamycin signalling modulates amino acid uptake by regulating transporter cell surface abundance in primary human trophoblast cells. J. Physiol.

[b51] Saucedo R, Zarate A, Basurto L, Hernandez M, Puello E, Galvan R (2011). Relationship between circulating adipokines and insulin resistance during pregnancy and postpartum in women with gestational diabetes. Arch. Med. Res.

[b52] Smith S, Francis R, Guilbert L, Baker PN (2002). Growth factor rescue of cytokine mediated trophoblast apoptosis. Placenta.

[b53] Uebel K, Pusch K, Gedrich K, Schneider KT, Hauner H, Bader BL (2014). Effect of maternal obesity with and without gestational diabetes on offspring subcutaneous and preperitoneal adipose tissue development from birth up to year-1. BMC Pregnancy Childbirth.

[b54] Verrey F (2003). System L: heteromeric exchangers of large, neutral amino acids involved in directional transport. Pflugers Arch.

[b55] Wang H, Huang W, Sugawara M, Devoe LD, Leibach FH, Prasad PD (2000). Cloning and functional expression of ATA1, a subtype of amino acid transporter A, from human placenta. Biochem. Biophys. Res. Commun.

[b56] Watkins KT, Dudrick PS, Copeland EM, Souba WW (1994). Interleukin-6 and dexamethasone work coordinately to augment hepatic amino acid transport. J. Trauma.

[b57] Xie X, Le L, Fan Y, Lv L, Zhang J (2012). Autophagy is induced through the ROS-TP53-DRAM1 pathway in response to mitochondrial protein synthesis inhibition. Autophagy.

[b58] Xu J, Zhao YH, Chen YP, Yuan XL, Wang J, Zhu H (2014). Maternal circulating concentrations of tumor necrosis factor-alpha, leptin, and adiponectin in gestational diabetes mellitus: a systematic review and meta-analysis. ScientificWorldJournal.

